# The pharmacokinetics effects of the MCAO model on senkyunolide I in pseudo germ-free rats after oral co-administration of Chuanxiong and warfarin

**DOI:** 10.3389/fphar.2025.1577757

**Published:** 2025-08-01

**Authors:** Haigang Li, Fang Li, Yi Zhou, Zhongjun Xiang, Bilan Zhou, Bin Lan, Xiaoling Ruan

**Affiliations:** ^1^ School of Chinese Medicine, Changsha Medical University, Changsha, China; ^2^ Hunan Provincial Key Laboratory of the Research and Development of Novel Pharmaceutical Preparations, Changsha Medical University, Changsha, China; ^3^ School of Pharmacy, Changsha Medical University, Changsha, China; ^4^ Department of Pharmacy, Changsha Health Vocational College, Changsha, China; ^5^ School of Medical Technology, Hunan University of Environment and Biology, Hengyang, China

**Keywords:** senkyunolide I, Chuanxiong, warfarin, pharmacokinetics, MCAO, pseudo germ-free

## Abstract

**Aim:**

To investigate the pharmacokinetics effects of a middle cerebral artery occlusion (MCAO) model on senkyunolide I (SEI) in pseudo germ-free rats after oral co-administration of Chuanxiong and warfarin.

**Methods:**

Rats were divided randomly into WC_H, WS_H, WC_HF, WS_HF, WC, WS, WC_F, and WS_F groups (W, warfarin; C, Chuanxiong; S, SEI; H, rats without an MCAO operation; F, pseudo germ-free). Pseudo germ-free rats were orally administered ciprofloxacin hydrochloride (50 mg/kg, twice a day) for 3 consecutive days, and the MCAO operation was carried out on the third day. Chuanxiong (10 g/kg) or SEI (7.5 mg/kg) and warfarin sodium (0.5 mg/kg) were orally administered only once on the fourth day. The plasma concentrations of SEI were determined by UPLC-MS/MS. Stool samples were collected for gut microbiota analyses. The coronal thin slices of the brain were dyed with triphenyl-2H-tetrazolium chloride.

**Results:**

Compared to the WC_H group, the AUC_0-t_ and C_max_ of the WC group increased 2.08- and 1.79-fold (*P < 0.001*), respectively. Compared with the WS_H group, the AUC_0-t_ and C_max_ of the WS group decreased by 6.24% and 22.64% (*P < 0.001*), respectively. Compared to the WC_HF group, the AUC_0-t_ and t_1/2z_ of the WC_F group decreased by 60.95% and 35.26% (*P < 0.01*), respectively. Compared with the WS_HF group, the AUC_0-t_ and C_max_ of the WS_F group decreased by 2.87% and 8.54% (*P < 0.001*), respectively. Compared to the WC_H group, the AUC_0-t_ and C_max_ of the WC_HF group increased by 85.03% and 82.48% (*P < 0.001*), respectively. Compared with the WS_H group, the AUC_0-t_ and C_max_ of the WS_HF group increased 1.40-and 2.43-fold (*P < 0.001*), respectively. Compared to the WC group, the AUC_0-t_ and t_1/2z_ of the WC_F group decreased by 45.81% and 79.44% (*P < 0.001*), respectively. Compared with the WS group, the AUC_0-t_ and t_1/2z_ of the WS_F group decreased by 35.86% and 79.74% (*P < 0.001*), respectively.

**Conclusion:**

Both the MCAO operation and the pseudo germ-free condition significantly changed the pharmacokinetics characteristics of SEI after oral co-administration with warfarin and Chuanxiong or SEI. It is a promising way to understand the potential pharmacokinetics of herb–drug interactions from the angle of the gut microbiota.

## Introduction

Thromboembolism is the third most common cause of cardiovascular mortality. The worldwide annual incidence rate is estimated to be approximately 115–269 per 100,000 people. The incidence is higher in men than women, increases with age, and approximately 30% of patients undergo recurrence within 10 years. Anticoagulation is the main treatment method (Malerba et al., 2024). Although direct oral anticoagulants (DOACs, such as apixaban and dabigatran) do not require dose adjustment and regular laboratory monitoring, warfarin is still one of the most commonly used oral anticoagulants for the treatment of thromboembolic diseases. Today, a well-managed warfarin treatment is an indispensable method of anticoagulation (Gao et al., 2022). Warfarin can maintain its effects for several days even if the patients stop taking it, whereas the DOACs do not have this merit (Li et al., 2016a).

There is increasing global acceptance of botanical drugs. Previous studies found that more than 80% of people take botanical drugs to maintain a healthy lifestyle in Africa and Asia (Singh and Zhao, 2017). More than 70% of people all over the world use botanical drugs as a supplementary or alternative method of treating cardiovascular diseases (Shaikh et al., 2020; Liang et al., 2023). It is estimated that approximately one-fourth of patients co-ingest warfarin and botanical drugs in Italy and Hong Kong (Cuzzolin et al., 2007; Wong et al., 2003). Compared to warfarin alone, concomitance with TCM had more advantages to prevent thromboembolic events evoked by atrial fibrillation with no higher occurrence rate of hemorrhage (Wang et al., 2017). Warfarin is usually prescribed with herbal formulas containing Chuanxiong in China, and these integrating methods were used most broadly for the treatment of thromboembolism in cardiovascular disorders (Feng et al., 2015; Zhang et al., 2016).

Our previous studies had demonstrated that Chuanxiong water extraction may obviously change the pharmacokinetics characteristics of warfarin in rats via the enterohepatic circulation pathway. As a single quality control biologically active metabolite of Chuanxiong (Chinese Pharmacopoeia Committee, 2020), ferulic acid cannot substitute for Chuanxiong to exert similar effects on warfarin to some extent (Li et al., 2016a; Li et al., 2016b). As the richest metabolite of water extraction of Chuanxiong (rhizomes of *Ligusticum chuanxiong*), senkyunolide I (SEI) exerts pharmacological functions, including antimigraine and antioxidant properties, which belong to the “facilitating blood circulation and dispersing blood stasis” of Chuanxiong (Wang et al., 2011; Wang et al., 2012; Xie et al., 2021).

The middle cerebral artery occlusion (MCAO) rodent model is frequently used to observe the changes of thromboembolism (Liu and McCullough, 2011; Chen et al., 2015). In this study, we investigated the pharmacokinetics effects of an MCAO model on SEI in pseudo germ-free rats and shed light on the potential functions of intestinal microbiota in the pharmacokinetics interactions between warfarin and Chuanxiong. These experimental results produced a promising method to understand the potential mechanisms of herb–drug interactions.

## Materials and methods

### Preparation and validation of Chuanxiong aqueous extract

The aqueous extract of Chuanxiong [rhizomes of Conioselinum anthriscoides ‘Chuanxiong’ (synonym: *Ligusticum chuanxiong* S.H.Qiu, Y.Q.Zeng, K.Y.Pan, Y.C.Tang & J.M.Xu), Lot, 20190202, Chengdu, China; voucher specimen no. 1908260, the Traditional Chinese Medicinal Herbarium, School of Changsha Medical University, Changsha, China] was prepared and validated as described previously (Li et al., 2023).

### Surgical procedures

All animal experiments were undertaken strictly following the guidelines for animal care and experimentation at Changsha Medical University and were approved by the Changsha Medical University Animal Ethics Committee (No. 20210318). Specific pathogen-free male SD rats (weight 180–220 g) were purchased from Hunan SJA Laboratory Animal Co., Ltd. (Changsha, China). They were placed in a well-ventilated room (four rats per cage) at a constant temperature (22°C–26°C, 45%–75% relative humidity) with a 12-h light/dark cycle in a specific pathogen-free environment. The MCAO surgical operation was performed under anesthesia and sterile conditions, as previously described (Li et al., 2023).

Sixty-four experimental rats were assigned randomly to the WC_H, WS_H, WC_HF, WS_HF, WC, WS, WC_F, and WS_F groups (W, warfarin; C, Chuanxiong; S, SEI; H, rats without MACO operation; F, pseudo germ-free, eight rats/group, Table 1). Pseudo germ-free rats were orally administered ciprofloxacin hydrochloride (50 mg/kg, twice a day) for 3 consecutive days. The MCAO operation was carried out on the third day. Chuanxiong (10 g/kg) or SEI (7.5 mg/kg) and warfarin sodium (0.5 mg/kg) were orally administered on the fourth day. SEI was obtained from Must Bio-technology Co., Ltd. (Chengdu, China). Warfarin sodium was ordered from Xinyi Jiufu Pharmaceutical Co., Ltd. (Shanghai, China). Ciprofloxacin hydrochloride was purchased from Meheco Topfond Pharma Co., Ltd. (Zhumadian, China). The animal dosages of the drugs were calculated using an equivalent dose conversion from human clinical dosages.

**TABLE 1:** Experimental animal groups.

Groups	Pseudo germ-free	MACO operation	Oral administration
WC	No	Yes	Warfarin and Chuanxiong
WC_F	Yes	Yes	Warfarin and Chuanxiong
WS	No	Yes	Warfarin and SEI
WS_F	Yes	Yes	Warfarin and SEI
WC_H	No	No	Warfarin and Chuanxiong
WC_HF	Yes	No	Warfarin and Chuanxiong
WS_H	No	No	Warfarin and SEI
WS_HF	Yes	No	Warfarin and SEI

Pseudo germ-free rats were orally administered ciprofloxacin hydrochloride (50 mg/kg, twice a day) for 3 consecutive days, and the MCAO operation was carried out on the third day. Chuanxiong (10 g/kg) or SEI (7.5 mg/kg) and warfarin sodium (0.5 mg/kg) were orally administered on the fourth day.

### Sample collection and preparation

One day (24 h) after the MCAO operation, and at the planned time points, blood samples were taken for analysis as previously described (Li et al., 2023). After the last blood sample was gathered, the abdomen of the experimental rat was opened, and a cecum stool sample was harvested and stored at −80°C until analysis. Taxonomic analyses were based on the bacterial 16S ribosomal RNA (rRNA) sequencing analyses, as previously described (Li et al., 2023).

### TTC staining

The animal’s brain was harvested and placed in a −20°C refrigerator for at least 30 min, and then the coronal brain slices of approximately 2 mm thickness were plunged into a dye solution [1% TTC (2,3,5-triphenyl-2H-tetrazolium chloride) in PBS, pH 7.4], and incubated in a 37°C constant temperature water bath for 20 min. After 24 h in cold 4% paraformaldehyde, the fixed brain slices were photographed for analysis.

### Instrumentation and analytical conditions

The determination of serum drug concentration was performed on a UPLC-MS/MS (ultra-performance liquid chromatography-tandem mass spectrometry) platform with multiple-reaction monitoring, as previously described (Li et al., 2023). DAS 2.1.1 software was used to accumulate pharmacokinetics data and analyze the related parameters.

### Statistical analysis

SAS software (Cary, North Carolina) was used for all statistical analyses. Data are expressed as mean ± standard deviation. The differences between normally distributed quantitative groups were determined with a Student’s t-test. Abnormally distributed variables were evaluated by the Mann–Whitney test. *P* < 0.05 was judged as statistically significant.

## Results

### Specificity of the UPLC-MS/MS method

**FIGURE 1:**
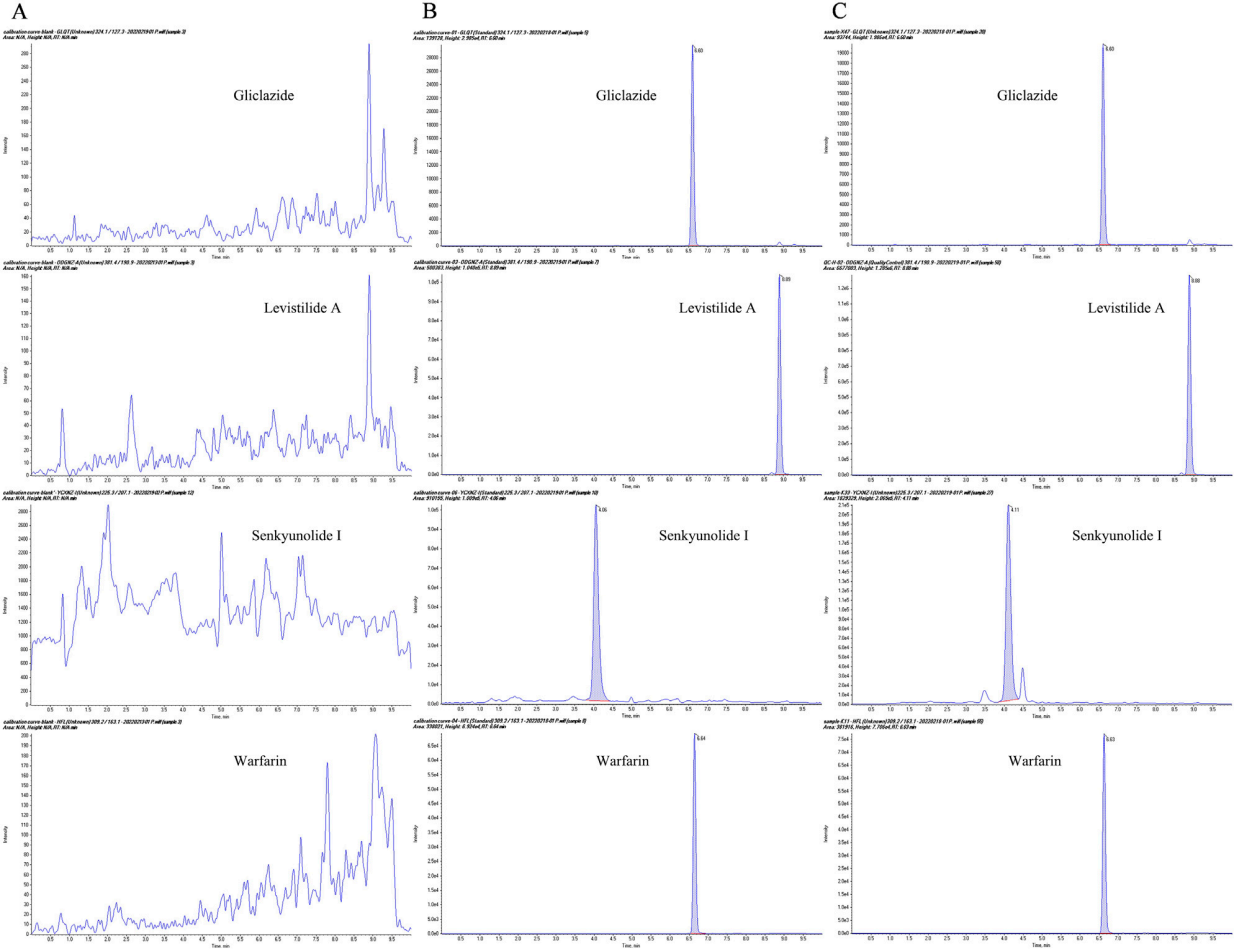
The representative multiple-reaction monitoring (MRM) chromatograms. **(A)** Blank plasma samples from MCAO rats; **(B)** Blank plasma samples spiked with gliclazide (IS), SEI, warfarin, and levistilide A; **(C)** Plasma from MCAO rats after co-administration of warfarin and Chuanxiong.

There was no interference between the retention times of the internal standard (IS, gliclazide) and SEI in plasma samples. The spectra of the IS and the SEI are displayed in Figure 1. The SEI linear regression equation was y = 0.0853 *x +* 0.7260 (r = 0.99767), the range of linearity was 5–10,000 ng/mL, and the LLOQ (lower limit of quantification) was 5 ng/mL, where *x* was the SEI concentration (ng/mL), *y* referred to the ratio of the peak area of SEI to that of IS, and the correlation coefficient (r) between predicted and real concentration was 0.99767. The precision and accuracy of the inter-day and intra-day values were all within the normal range (Table 2).

**TABLE 2:** The intra-day and inter-day precision of SEI in MCAO rat plasma samples.

	Spiked concentration	Intra-day			Inter-day
	(ng/mL)	Measured (ng/mL)	RSD%	Measured (ng/mL)	RSD%
	10	10.32 ± 0.31	3.00	9.73 ± 0.46	4.73
SEI	100	97.66 ± 4.70	4.81	103.76 ± 6.51	6.26
	1,000	1,021.78 ± 67.42	6.60	1,083.02 ± 73.25	6.76
	10,000	10,129.37 ± 808.25	7.98	9,838.05 ± 805.30	8.19

### TTC results for the MCAO model

**FIGURE 2:**
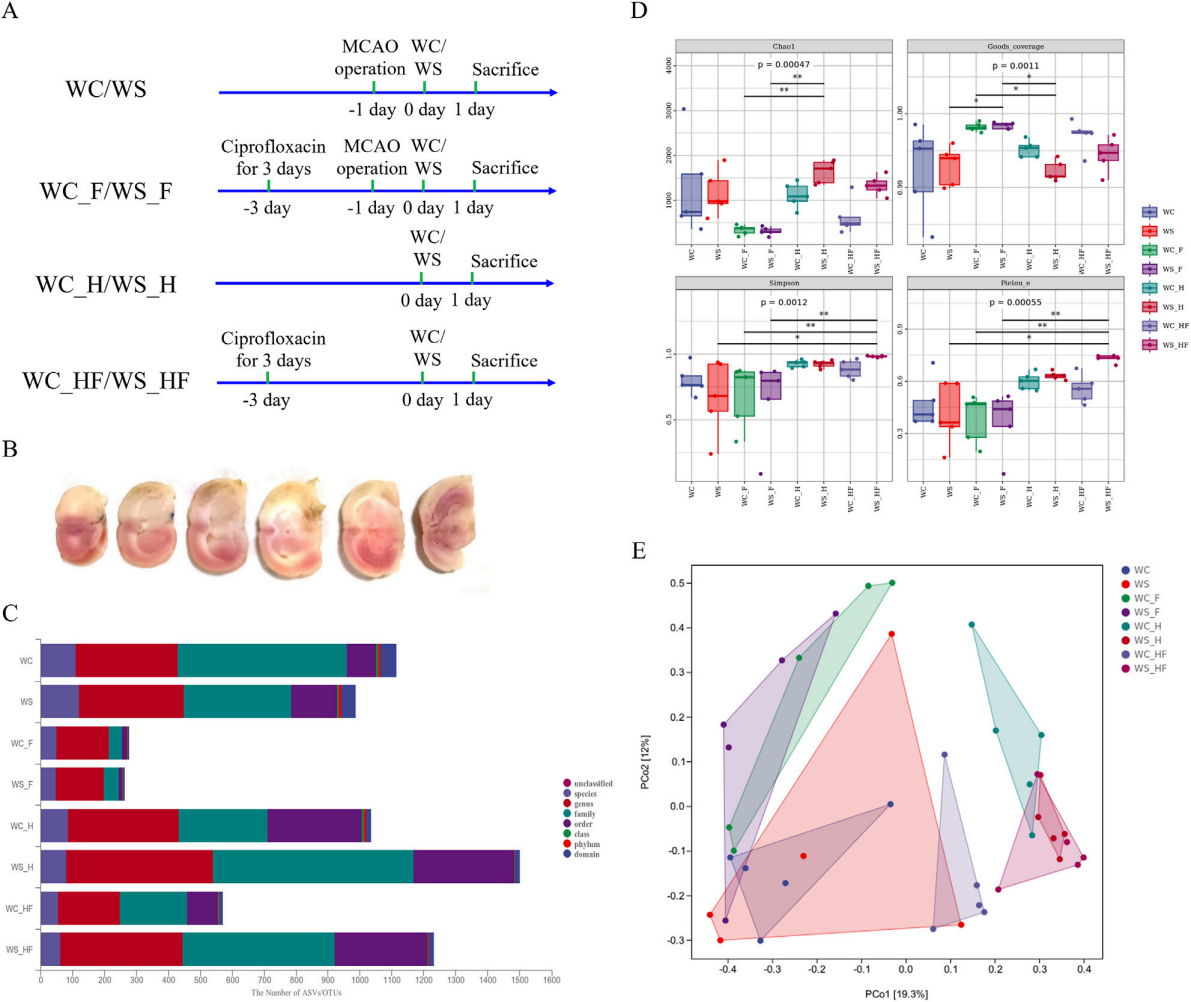
The TTC staining of the brains of MCAO rats and the results of 16S rRNA sequencing of the gut microbiota. **(A)** Animal experimental design; **(B)** the TTC staining of MCAO rats; **(C)** the ASVs/OUTs number at each taxonomic level of phylum, class, order, family, genus, and species; **(D)** alpha boxplot; **(E)** distance matrix and PCoA. WC, MCAO rats co-administered warfarin and Chuanxiong; WS, MCAO rats co-administered warfarin and SEI; WC_F, pseudo germ-free MCAO rats co-administered warfarin and Chuanxiong; WS_F, pseudo germ-free MCAO rats co-administered warfarin and SEI; WC_H, healthy rats co-administered warfarin and Chuanxiong; WS_H, healthy rats co-administered warfarin and SEI; WC_HF, healthy pseudo germ-free rats co-administered warfarin and Chuanxiong; WS_HF, healthy pseudo germ-free rats co-administered warfarin and SEI.

The volume of TTC-stained cerebral infarction is displayed in Figure 2B. The white region of the TTC-stained brain slice indicates infarction (the occluded lateral brain), and the red region represents non-infarction. These results confirm that the MCAO rat model was successfully established.

### Gut microbiota 16S rRNA sequencing

The 16S rRNA gene sequencing was used to evaluate the pharmacokinetics effects of the MCAO model on SEI in pseudo germ-free rats after the oral co-administration of Chuanxiong and warfarin. The microbiota diversity is displayed in a box plot of the alpha diversity of the gut microbiota. Species taxonomy annotation was used to determine the differentially abundant bacterial taxa among all groups (Figure 2).

**FIGURE 3:**
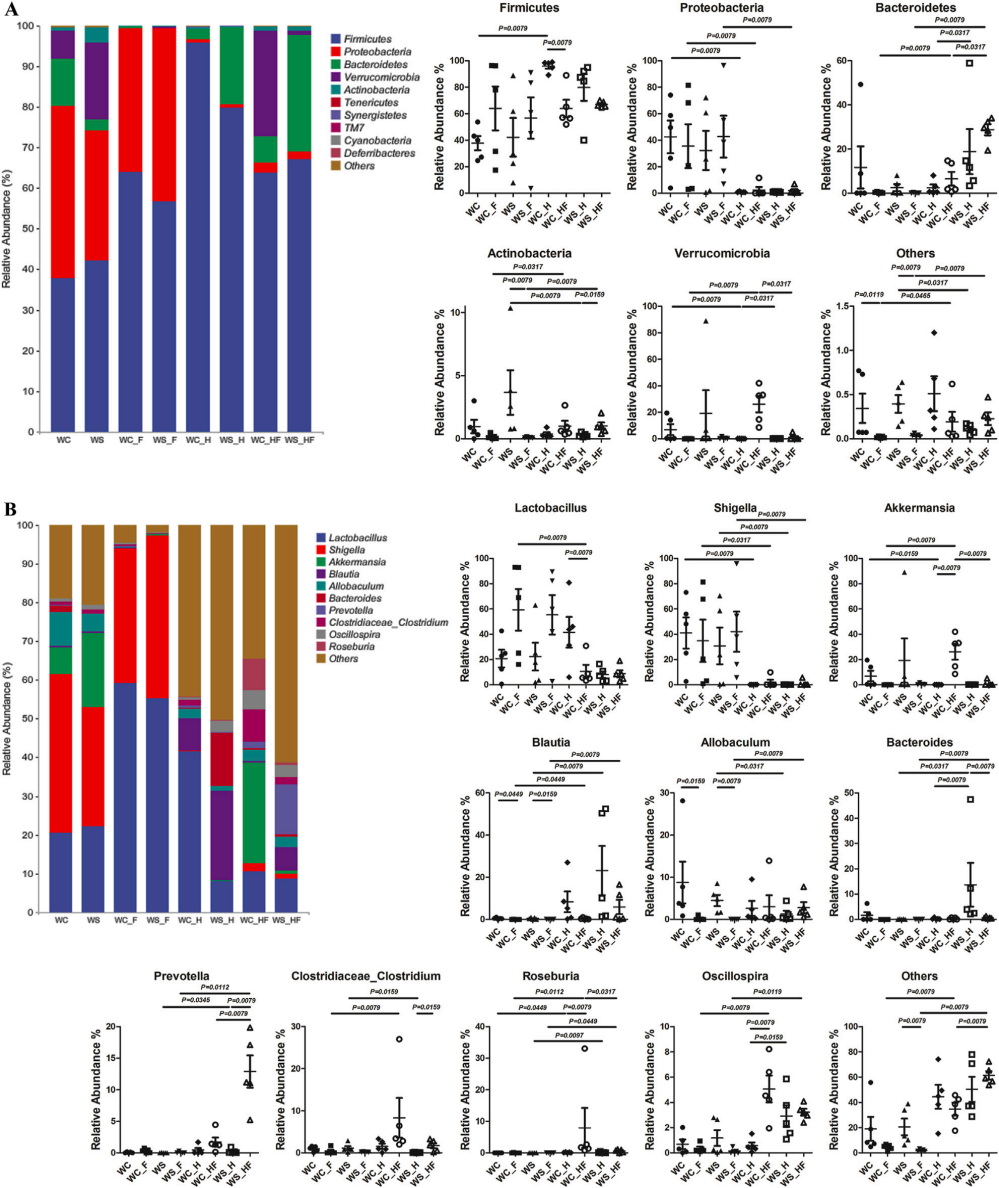
The relative abundance of gut microbiota compositions at the phylum level **(A)** and genus level **(B)**. WC, MCAO rats co-administered warfarin and Chuanxiong; WS, MCAO rats co-administered warfarin and SEI; WC_F, pseudo germ-free MCAO rats co-administered warfarin and Chuanxiong; WS_F, pseudo germ-free MCAO rats co-administered warfarin and SEI; WC_H, healthy rats co-administered warfarin and Chuanxiong; WS_H, healthy rats co-administered warfarin and SEI; WC_HF, healthy pseudo germ-free rats co-administered warfarin and Chuanxiong; WS_HF, healthy pseudo germ-free rats co-administered warfarin and SEI.

The microbiota structure and relative abundance are shown in Figure 3. The relative abundance of *Firmicutes* was the highest ratio of the total bacteria at the phylum level, reaching 95.91% and 79.67% in healthy rats with normal flora (WC_H and WS_H groups). *Proteobacteria* and *Firmicutes* were the two major phyla in the WC group, with ratios of 79.93% (42.41% and 37.52%, respectively), and in the WS group, with ratios of 74.13% (32.21% and 41.92%, respectively). In total, these ratios increased to 99.39% (35.76% and 63.63%, respectively) and 99.38% (42.75% and 56.63%, respectively) in the pseudo germ-free WC_F and WS_F groups. The ratio of *Proteobacteria* among the three healthy groups (WC_H, WC_HF, and WS_HF) was lower than that of the three MCAO groups (WC, WC_F, and WS_F).

The genus *Lactobacillus* had the highest relative abundance in the WC_H, WC_F, and WS_F groups (reaching 41.51%, 59.16%, and 55.14%, respectively). The ratio of the *Shigella* genus in the four MCAO groups (WC, WC_F, WS, and WS_F) was higher than in the other four healthy groups (WC_H, WC_HF, WS_H, and WS_HF), while *Roseburia* showed an exactly opposite tendency at a relatively low level. The ratio of the *Blautia* genus in the three MCAO groups (WC_F, WS, and WS_F) was less than that in the other three healthy groups with no MCAO operation (WC_HF, WS_H, and WS_HF).

### Pharmacokinetics study

**FIGURE 4:**
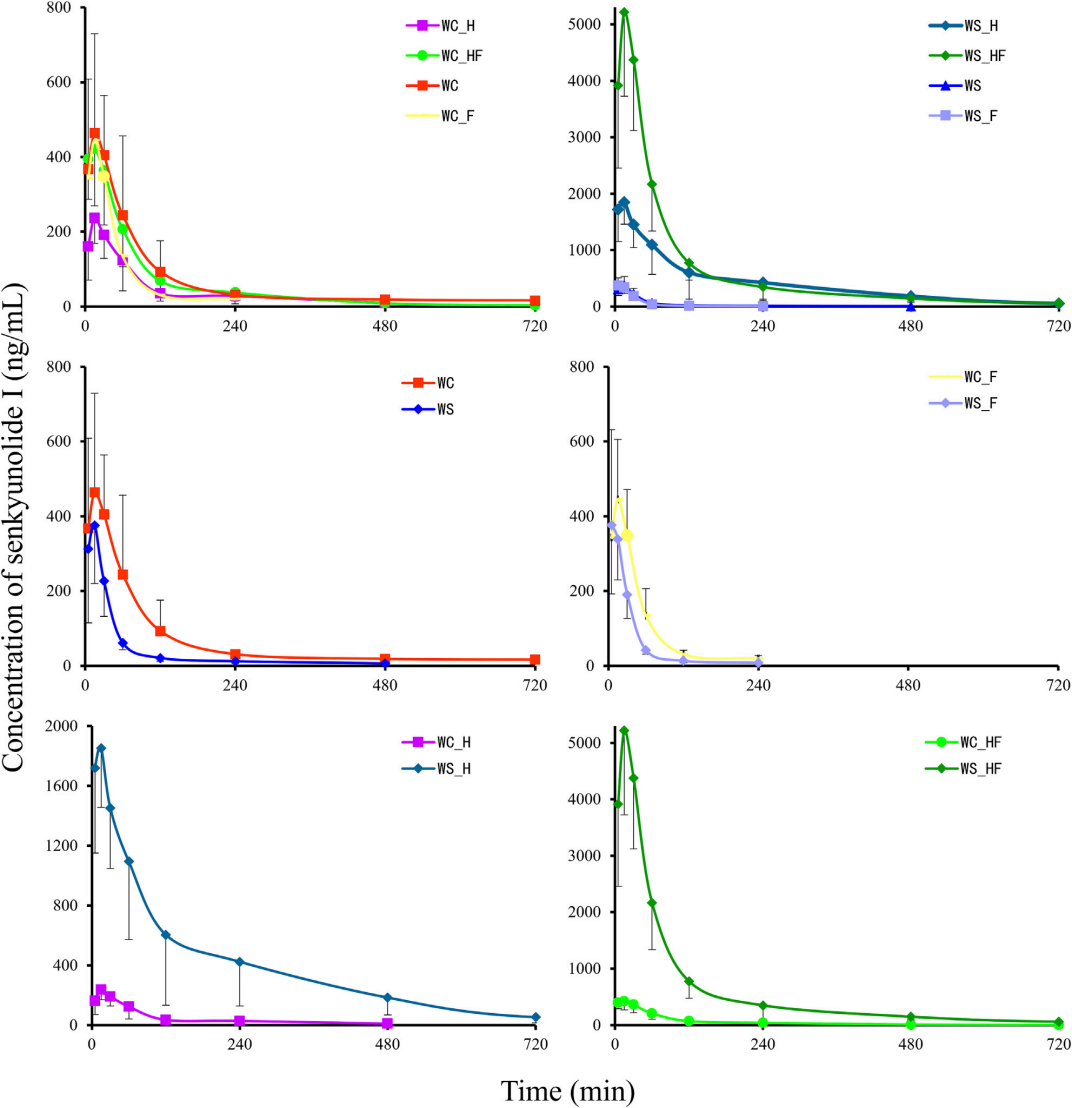
The pharmacokinetics effects of the MCAO model on SEI in pseudo germ-free rats (*n* = 8). The MCAO model and pretreatment with ciprofloxacin both change the bioavailability of SEI. WC, MCAO rats co-administered warfarin and Chuanxiong; WS, MCAO rats co-administered warfarin and SEI; WC_F, pseudo germ-free MCAO rats co-administered warfarin and Chuanxiong; WS_F, pseudo germ-free MCAO rats co-administered warfarin and SEI; WC_H, healthy rats co-administered warfarin and Chuanxiong; WS_H, healthy rats co-administered warfarin and SEI; WC_HF, healthy pseudo germ-free rats co-administered warfarin and Chuanxiong; WS_HF, healthy pseudo germ-free rats co-administered warfarin and SEI.

Figure 4 shows that there were more differences in the concentration-time profiles of plasma SEI among all groups. The non-compartmental pharmacokinetics parameters are displayed in Table 3. In contrast to the WC_H group, the C_max_ and AUC_0-t_ of the WS_H group increased 8.68-fold (*P < 0.001*) and 15.53-fold (*P < 0.001*). The relative abundance of *Bacteroides* in the WS_H group (3.94%, median) was significantly higher than in the WC_H group (0.13%, median, *P = 0.0079*). The median relative abundance of *Oscillospira* increased from 0.46% to 2.33% (*P = 0.0159*). In contrast to the WC_HF group, the C_max_ and AUC_0-t_ of the WS_HF group increased 11.58-fold (*P < 0.001*) and 11.72-fold (*P < 0.001*), respectively. The relative abundance of *Akkermansia* in the WS_HF group (0.01%, median) was much lower than that of the WC_HF group (32.48%, median, *P = 0.0079*), while the median relative abundance of *Prevotella* increased from 1.31% to 11.19% (*P = 0.0079*). At the same time, the median relative abundance of *Roseburia* decreased from 1.74% to 0.73% (*P = 0.0317*). In contrast to the WC group, the t_1/2z_ and AUC_0-t_ of the WS group decreased by 45.22% (*P < 0.001*) and 53.42% (*P < 0.001*), respectively. In contrast to the WC_F group, the t_1/2z_ and AUC_0-t_ of the WS_F group decreased by 46.02% (*P < 0.001*) and 44.87% (*P < 0.001*), respectively, and no significant difference was found in the constitution of microbiota.

**TABLE 3:** The pharmacokinetics parameters of SEI (mean ± SD, *n* = 8).

Groups	AUC_(0-t)_ (ng/mL·min)	MRT_(0-t)_ (min)	t_1/2z_ (min)	T_max_ (min)	C_max_ (ng/mL)
WC	48,181.5 ± 26,412.0	174.2 ± 82.7	195.5 ± 91.5	36.6 ± 47.0	443.6 ± 280.3
WC_F	26,109.0 ± 5,466.9 ^aa^	50.3 ± 10.5 ^aa^	40.2 ± 16.1^aa^	14.0 ± 10.2 ^aa^	531.0 ± 230.4
WS	22,441.5 ± 4,328.8 ^aa^	67.1 ± 17.6 ^aa^	107.1 ± 57.0 ^aa^	14.0 ± 10.2 ^aa^	485.5 ± 147.5
WS_F	14,394.8 ± 2,978.0 ^bb^ ^ee^	33.3 ± 9.9 ^bb^ ^ee^	21.7 ± 3.6 ^bb^ ^ee^	7.0 ± 4.5 ^bb^ ^ee^	445.7 ± 151.1^e^
WC_H	23,151.7 ± 9,285.5 ^aa^	90.3 ± 32.4 ^aa^	93.4 ± 42.5 ^aa^	19.0 ± 10.8^a^	247.2 ± 69.1^aa^
WC_HF	42,838.0 ± 17,106.5 ^cc^ ^ee^	107.2 ± 23.3 ^cc^ ^ee^	114.0 ± 42.4^c^ ^ee^	13.0 ± 4.5 ^cc^	451.1 ± 162.5 ^cce^
WS_H	359,444.7 ± 205,432.1^bb^ ^cc^	244.8 ± 105.8 ^bb^ ^cc^	247.7 ± 122.2 ^bbc^	25.0 ± 21.5 ^bb^	2,144.5 ± 578.3 ^bb^ ^cc^
WS_HF	502,135.9 ± 134,214.8 ^dd ff gg^	161.0 ± 37.7 ^dd ff gg^	229.3 ± 78.2 ^ff^ ^gg^	15.0 ± 0.0 ^dd ff gg^	5,221.4 ± 1,496.6 ^dd ff gg^

^a^, compared to the WC group, p < 0.05.

^aa^, compared to the WC group, p < 0.01.

^bb^, compared to the WS group, p < 0.01.

^c^, compared to the WC_H group, p < 0.05.

^cc^, compared to the WC_H group, p < 0.01.

^dd^, compared to the WS_H group, p < 0.01.

^e^, compared to the WC_F group, p < 0.05.

^ee^, compared to the WC_F group, p < 0.01.

^ff^, compared to the WC_HF, group, p < 0.01.

^gg^, compared to the WS_F group, p < 0.01.

WC, MCAO, rats co-administered warfarin and Chuanxiong; WS, MCAO, rats co-administered warfarin and SEI; WC_F, pseudo germ-free MCAO, rats co-administered warfarin and Chuanxiong; WS_F, pseudo germ-free MCAO, rats co-administered warfarin and SEI; WC_H, healthy rats co-administered warfarin and Chuanxiong; WS_H, healthy rats co-administered warfarin and SEI; WC_HF, healthy pseudo germ-free rats co-administered warfarin and Chuanxiong; WS_HF, healthy pseudo germ-free rats co-administered warfarin and SEI.

In contrast to the WC_H group, the C_max_ and AUC_0-t_ of the WC group increased 1.79-fold (*P < 0.001*) and 2.08-fold (*P < 0.001*), respectively. The median relative abundances of *Shigella* and *Akkermansia* in the WC group (48.08% and 0.38%, respectively) were significantly higher than that of the WC_H group (0.12%, *P = 0.0079,* and 0.01%, *P = 0.0159,* respectively), while the median relative abundance of *Romboutsia* decreased from 0.12% to zero (*P = 0.0449*). In contrast to the WS_H group, the C_max_ and AUC_0-t_ of the WS group decreased by 6.24% (*P < 0.001*) and 22.64% (*P < 0.001*), respectively. The relative abundance of *Shigella* in the WS group (23.69%, median) was significantly higher than that of the WS_H group (0.12%, median, *P = 0.0079*), while the median relative abundances of *Blautia* and *Bacteroides* decreased from 10.01% to 3.94%–0.31% (*P = 0.0079*) and 0.01% (*P = 0.0079*), respectively.

In contrast to the WC_HF group, the t_1/2z_ and AUC_0-t_ of the WC_F group decreased by 35.26% (*P = 0.003*) and 60.95% (*P < 0.001*), respectively. The median relative abundances of *Lactobacillus* and *Shigella* in the WC_F group (69.14% and 20.45%, respectively) were significantly higher than those of the WC_HF group (5.48%, *P = 0.0317*, and 0.03%, *P = 0.0317*, respectively). The median relative abundance of *Akkermansia* decreased from 32.48% to 0.38% (*P = 0.0079*). At the same time, *Clostridiaceae_Clostridium*, *Oscillospira*, and *Roseburia* all decreased (*P < 0.05*). In contrast to the WS_HF group, the C_max_ and AUC_0-t_ of the WS_F group decreased by 8.54% (*P < 0.001*) and 2.87% (*P < 0.001*), respectively. The relative abundance of *Shigella* in the WS_F group (38.05%, median) was significantly higher than that of the WS_HF group (0.08%, median, *P = 0.0079*), while *Blautia*, *Allobaculum*, *Bacteroides, Prevotella*, *Oscillospira*, and *Roseburia* decreased (*P < 0.05*).

In contrast to the WC_H group, the C_max_ and AUC_0-t_ of the WC_HF group increased by 82.48% (*P < 0.001*) and 85.03% (*P < 0.001*), respectively. The relative abundance of genus *Lactobacillus* in the WC_HF group (5.48%, median) was less than that of the WC_H group (43.71%, median, *P = 0.0317*). The median relative abundance of *Akkermansia* increased from 0.01% to 32.48% (*P = 0.0079*), and *Oscillospira* and *Roseburia* both slightly increased (*P = 0.0079*). In contrast to the WS_H group, the C_max_ and AUC_0-t_ of the WS_HF group increased 2.43-fold (*P < 0.001*) and 1.40-fold (*P < 0.001*), respectively. The median relative abundances of *Clostridiaceae_Clostridium* and *Prevotella* in the WS_HF group (1.86% and 11.19%) were significantly higher than those of the WS_H group (0.06%, *P = 0.0159*, and 0.08%, *P = 0.0079*, respectively), while *Bacteroides* decreased from 3.94% to 0.56% (*P = 0.0079*).

In contrast to the WC group, the t_1/2z_ and AUC_0-t_ of the WC_F group decreased by 45.81% (*P < 0.001*) and 79.44% (*P < 0.001*), respectively. The median relative abundances of *Blautia* and *Allobaculum* in the WC_F group (almost zero) were less than those of the WC group (0.44%, *P = 0.0449*, and 3.79%, *P = 0.0159*, respectively). In contrast to the WS group, the t_1/2z_ and AUC_0-t_ of the WS_F group decreased by 79.74% (*P < 0.001*) and 35.86% (*P < 0.001*), respectively. The median relative abundances of *Allobaculum* and *Blautia* decreased from 4.78% to 0.31% to almost zero (*P < 0.05*).

## Discussion

Our published studies have shown that SEI is the most abundant metabolite in a water extraction of Chuanxiong, and SEI presents as a relatively complete and detectable concentration-time curve after intra-gastric administration of Chuanxiong water extraction in rat plasma (Li et al., 2018; Li et al., 2023). Warfarin significantly enhances the bioavailability of SEI (in rats, oral co-administered Chuanxiong and warfarin), partly due to the enterohepatic circulation (Li et al., 2018). As another main bioactive metabolite in Chuanxiong, ligustilide is fragile and prone to being metabolized into SEI (Zuo et al., 2011). Glucuronidation, hydrolysis, and glutathione conjugation are the three major metabolic pathways of SEI in rats (Ma et al., 2015). The greater amount of glucuronidation underlies a higher potential for the hepatoenteral circulation process (Liu et al., 2023). After entering the intestinal tract, the glucuronidation metabolites excreted by the bile duct are hydrolyzed by the special microbiota into free aglycones. SEI is absorbed more quickly in the digestive tract, and almost 90% of that is secreted from the bile duct (Ma et al., 2015).

As a broad-spectrum antibiotic, ciprofloxacin was introduced into our present research as a pseudo germ-free tool to validate the pharmacokinetics of the interaction of Chuanxiong and warfarin from the angle of microbiota. As depicted in Figure 4, the MCAO operation and pretreatment of ciprofloxacin markedly changed the constitution and relative abundance of THE gut microbiota.

Compared to THE co-administration of warfarin and Chuanxiong, the pharmacokinetics parameters of the co-administration of warfarin and SEI presented an exactly opposite tendency between rats with the MCAO operation and healthy rats, regardless of whether the rats were pretreated with ciprofloxacin. As displayed in Table 2 and Figure 4, compared to the WC group, the AUC of the WS group decreased by 53.42%. Compared to the WC_F group, the AUC of the WS_F group significantly decreased by 44.87%, but there were no obvious differences between the two in terms of microbiota constitution. Compared with the WC_H group, there was a tremendous increase in the C_max_ and AUC of the WS_H group, and the relative abundances of *Bacteroides* and *Oscillospira* in the WS_H group were notably higher than those of the WC_H group. In contrast with the WC_HF group, there was also a substantial increase in the C_max_ and AUC of the WS_HF group. The relative abundance of *Akkermansia* in the WS_HF group was lower than that of the WC_HF group, but that of *Prevotella* increased.

The opposite tendency in the pharmacokinetics effects might be due to the MCAO operation, which obviously affects the composition of the intestinal microbiota, such as the relative prevalence of *Bacteroidetes* and fewer species (Singh and Zhao, 2017), and the loss of integrity of the gut mucosal barrier (Stanley et al., 2016). Loss of intestinal integrity would further reduce the absorption of SEI.

In a healthy rat liver, competing for the same enzymes and transporters as warfarin, the less SEI is excreted into the bile, the more will enter into the blood. Similarly, such competition might be generated mostly in the digestive tract of MCAO rats, and this situation would further reduce the uptake of SEI. Additionally, the MCAO operation would involve some complicating factors and affect the SEI disposition process (Zhang et al., 2019). These conflicts might provide a potential chance to investigate the pharmacokinetics mechanism of SEI in rats.

Depending on the differences between the WC and WC_H groups, the MCAO operation significantly enhanced the bioavailability of SEI when Chuanxiong was co-ingested with warfarin. However, the state of pseudo germ-free (pretreatment with ciprofloxacin) displayed an opposite tendency to the comparison between the WC_F and WC_HF groups: the MCAO operation significantly decreased the bioavailability of SEI. Gut microbiota dysbiosis induced by ciprofloxacin produced an obvious influence on the pharmacokinetics interaction of warfarin and Chuanxiong. Intestinal microbiota could intervene in the pharmacokinetics interactions of warfarin and Chuanxiong to some extent. The comparisons between the WS and WS_H groups and between the WS_F and WS_HF groups suggest that the MCAO operation and pretreatment with ciprofloxacin both significantly decreased the bioavailability of SEI when co-administered with warfarin.

These results indicated that multiple metabolites in Chuanxiong could dramatically affect the pharmacokinetics characteristics of SEI in rats. As some studies have reported, the systemic exposure of a single monomer can create remarkably more influence by co-administering the other monomer than that of the extract of the botanical drug in healthy rats (Wang et al., 2022; Yang et al., 2019). Some other metabolites of Chuanxiong might have a negative impact on the systemic exposure of SEI and might subsequently degrade its bioavailability. Compared to the single bioactive metabolite (SEI), the botanical drug (Chuanxiong) would produce a more gentle and stable influence on the pharmacokinetics of drug–drug interactions (Li et al., 2023).

Compared to MCAO rats, whether Chuanxiong or SEI is co-administered with warfarin, rats with no operation experience the opposite pharmacokinetics parameters of SEI, depending on whether they were pretreated with ciprofloxacin or not. Comparisons between the WC_H and WC_HF groups and the comparison between the WS_H and WS_HF groups suggest that pretreatment with ciprofloxacin significantly increased the bioavailability of SEI. Our present research showed that the relative abundance of the *Akkermansia, Oscillospira*, and *Roseburia* genera in the WC_HF group was significantly higher than that of the WC_H group, and the *Lactobacillus* genus showed an exactly opposite tendency. The relative abundance of the *Prevotella* and *Clostridiaceae_Clostridium* genera in the WS_HF group was significantly higher than that of the WS_H group, and *Bacteroides* showed an exactly opposite tendency.

According to the comparisons between the WC and WC_F groups and the comparison between the WS and WS_F groups, pretreatment with ciprofloxacin decreased the bioavailability of SEI. After oral administration, SEI can penetrate the blood–brain barrier (He et al., 2012), and the MCAO operation can damage the integrity of the rat blood–brain barrier (Hu et al., 2024). More drug penetrates the brain, and more are absorbed from the digestive tract. Antibiotic treatment can improve stroke outcomes and reduce neuroinflammation in the MCAO brain (Kurita et al., 2020). The present research manifested that the relative abundance of the *Shigella* genus in the MCAO groups was higher than in the other four groups with no MCAO operation (WC_H, WC_HF, WS_H, and WS_HF), and *Roseburia* displayed an exactly opposite tendency at a relatively low level. The relatively higher abundance of *Shigella* can decrease the integrity of the rat blood–brain barrier via down-regulating the levels of occludin, claudin-5, and ZO-1 in the brain (Chen et al., 2019).

Although we verified that MCAO operation and pretreatment of ciprofloxacin affected the pharmacokinetics characteristics of SEI in rats after co-digestion of Chuanxiong or SEI with warfarin, it is not clear exactly how it works. We lack adequate evidence about the diverse enzyme/transporter pathways in the pharmacokinetics process of Chuanxiong, although other published studies showed that these processes may involve monocarboxylic acid transporters, cytochrome P450s (CYPs), sulfotransferases, and UDP-glucuronosyltransferases (Poquet et al., 2008; Su et al., 2023). It is reasonable to presume that the pharmacokinetics of herb–drug interactions between Chuanxiong and warfarin relate to more factors than only the above indexes. Further studies on the pharmacokinetics of SEI and other main bioactive metabolites of Chuanxiong should be performed.

## Conclusion

Both the MCAO operation and the use of pseudo germ-free conditions (pretreatment with ciprofloxacin) significantly affected the disposition of SEI in rats after the gastric co-digestion of warfarin and Chuanxiong or SEI. The pharmacokinetics process of SEI is significantly affected by the MCAO operation, which is partly attributed to gut microbiota, after the oral co-administration of Chuanxiong (or SEI) and warfarin. The present research and our previous studies verified that the pharmacokinetics of herb–drug interactions are intricate.

## Data Availability

The original contributions presented in the study are publicly available. This data can be found at the NCBI repository, accession numbers PRJNA873289 and PRJNA1293152.

## Abbreviations

AUC, area under the curve of concentration-time; CYP, cytochrome P450; C_max_, maximum plasma concentration; DOACs, direct oral anticoagulants; IS, internal standard; LEfSe, linear discriminant analysis effect size algorithm; LLOQ, lower limit of quantification; MCAO, middle cerebral artery occlusion; MRM, multiple-reaction monitoring; MRT, mean residence time; PBS, phosphate-buffered saline; SD, Sprague–Dawley; rRNA, ribosomal RNA; SEI, senkyunolide I; t_1/2_, half-life; TCM, traditional Chinese medicine; T_max_, time to reach the maximum concentration; TTC, 2,3,5-triphenyl-2H-tetrazolium chloride; UPLC-MS/MS, ultra-performance liquid chromatography coupled to tandem mass spectrometry; WC_H, healthy rats co-administered warfarin and Chuanxiong; WS_H, healthy rats co-administered warfarin and SEI; WC_HF, healthy pseudo germ-free rats co-administered warfarin and Chuanxiong; WS_HF, healthy pseudo germ-free rats co-administered warfarin and SEI; WC, MCAO rats co-administered warfarin and Chuanxiong; WS, MCAO rats co-administered warfarin and SEI; WC_F, pseudo germ-free MCAO rats co-administered warfarin and Chuanxiong; WS_F, pseudo germ-free MCAO rats co-administered warfarin and SEI.

## References

[B1] Chen R. Wu P. Cai Z. Fang Y. Zhou H. Lasanajak Y. (2019). Puerariae Lobatae Radix with Chuanxiong rhizoma for treatment of cerebral ischemic stroke by remodeling gut microbiota to regulate the brain-gut barriers. J. Nutr. Biochem. 65, 101–114. 10.1016/j.jnutbio.2018.12.004 30710886

[B2] Chen Y. Zhu W. Zhang W. Libal N. Murphy S. J. Offner H. (2015). A novel mouse model of thromboembolic stroke. J. Neurosci. Methods. 256, 203–211. 10.1016/j.jneumeth.2015.09.013 26386284 PMC4651806

[B3] Chinese Pharmacopoeia Committee (2020). Chinese Pharmacopoeia. Beijing: China Medical Science Press.

[B4] Cuzzolin L. Francini-Pesenti F. Zaffani S. Brocadello F. Pengo V. Bassi A. (2007). Knowledges about herbal products among subjects on warfarin therapy and patient-physician relationship: a pilot study. Pharmacoepidemiol. Drug Saf. 16 (9), 1014–1017. 10.1002/pds.1446 17615601

[B5] Feng B. Wang J. L. Zhuang P. W. Liu D. Tong Y. L. Zhang Y. J. (2015). Interaction of Shunaoxin dripping pill and warfarin sodium on anticoagulant. Chin. Tradit. Herb. Drugs, 2445–2448. 10.1360/ze2011-41-6-725

[B6] Gao X. Huang D. Hu Y. Chen Y. Zhang H. Liu F. (2022). Direct oral anticoagulants vs. vitamin K antagonists in atrial fibrillation patients at risk of falling: a meta-analysis. Front. Cardiovasc. Med. 9, 833329. 10.3389/fcvm.2022.833329 35615562 PMC9124845

[B7] He C. Y. Wang S. Feng Y. Liang S. Lin X. Xu D. S. (2012). Pharmacokinetics, tissue distribution and metabolism of senkyunolide I, a major bioactive component in Ligusticum chuanxiong Hort. (Umbelliferae). J. Ethnopharmacol. 142, 706–713. 10.1016/j.jep.2012.05.047 22668502

[B8] Heneghan C. Aronson J. (2020). Extending anticoagulation treatment for unprovoked venous thromboembolism. BMJ Evid. Based Med. 25 (5), 184–186. 10.1136/bmjebm-2019-111252 31771946

[B9] Hu X. Dong J. Geng P. Sun Y. Du W. Zhao X. (2024). Nicotine treatment ameliorates blood-brain barrier damage after acute ischemic stroke by regulating endothelial scaffolding protein Pdlim5. Transl. Stroke Res. 15, 672–687. 10.1007/s12975-023-01158-0 37233908

[B10] Kurita N. Yamashiro K. Kuroki T. Tanaka R. Urabe T. Ueno Y. (2020). Metabolic endotoxemia promotes neuroinflammation after focal cerebral ischemia. J. Cereb. Blood Flow. Metab. 40 (12), 2505–2520. 10.1177/0271678X19899577 31910709 PMC7820690

[B11] Li H. Jiang Y. Wang Y. Lv H. Xie H. Yang G. (2018). The effects of warfarin on the pharmacokinetics of senkyunolide I in a rat model of biliary drainage after administration of Chuanxiong. Front. Pharmacol. 9, 1461. 10.3389/fphar.2018.01461 30631279 PMC6315196

[B12] Li H. Wang Y. Fan R. Lv H. Sun H. Xie H. (2016a). The effects of ferulic acid on the pharmacokinetics of warfarin in rats after biliary drainage. Drug Des. Devel. Ther. 10, 2173–2180. 10.2147/DDDT.S107917 PMC4940002 27462142

[B13] Li H. Zhang C. Fan R. Sun H. Xie H. Luo J. (2016b). The effects of Chuanxiong on the pharmacokinetics of warfarin in rats after biliary drainage. J. Ethnopharmacol. 193, 117–124. 10.1016/j.jep.2016.08.005 27497635

[B14] Li H. Zhou Y. Liao L. Tan H. Li Y. Li Z. (2023). Pharmacokinetics effects of chuanxiong rhizoma on warfarin in pseudo germ-free rats. Front. Pharmacol. 13, 1022567. 10.3389/fphar.2022.1022567 36686675 PMC9849362

[B15] Liang D. Guan Q. Huang M. He Y. Ou Y. Chen M. (2023). Changing trends of disease burden of stroke from 1990 to 2019 and its predictions among the Chinese population. Front. Neurol. 14, 1255524. 10.3389/fneur.2023.1255524 37869143 PMC10588696

[B16] Liu F. McCullough L. D. (2011). Middle cerebral artery occlusion model in rodents: methods and potential pitfalls. J. Biomed. Biotechnol. 2011, 464701. 10.1155/2011/464701 21331357 PMC3035178

[B17] Liu Y. Li H. Wang X. Huang J. Zhao D. Tan Y. (2023). Anti-alzheimers molecular mechanism of icariin: insights from gut microbiota, metabolomics, and network pharmacology. J. Transl. Med. 21, 277. 10.1186/s12967-023-04137-z 37095548 PMC10124026

[B18] Ma C. Xiong Y. K. Ma S. Y. Fu Z. Li J. Liang S. (2015). Urine and bile excretion of senkyunolide Ⅰ in rats. China J. Exp. Traditional Med. Formulae 19, 75–79. 10.14725/gjicmwm.v2n2a387

[B19] Malerba S. A. Fumagalli R. M. Ay C. Cesarman-Maus G. De Paula E. V. Dumantepe M. (2024). Availability of medical and endovascular therapies for venous thromboembolism: a global survey for World Thrombosis Day. J. Thromb. Haemost. 22 (1), 255–262. 10.1016/j.jtha.2023.10.002 37838241

[B20] Poquet L. Clifford M. N. Williamson G. (2008). Transport and metabolism of ferulic acid through the colonic epithelium. Drug Metab. Dispos. 36, 190–197. 10.1124/dmd.107.017558 17954526

[B21] Shaikh A. S. Thomas A. B. Chitlange S. S. (2020). Herb-drug interaction studies of herbs used in treatment of cardiovascular disorders-A narrative review of preclinical and clinical studies. Phytother. Res. 34, 1008–1026. 10.1002/ptr.6585 31908085

[B22] Singh A. Zhao K. (2017). Herb-drug interactions of commonly used Chinese medicinal herbs. Int. Rev. Neurobiol. 135, 197–232. 10.1016/bs.irn.2017.02.010 28807159

[B23] Stanley D. Mason L. J. Mackin K. E. Srikhanta Y. N. Lyras D. Prakash M. D. (2016). Translocation and dissemination of commensal bacteria in post-stroke infection. Nat. Med. 22 (11), 1277–1284. 10.1038/nm.4194 27694934

[B24] Su Y. N. Wang M. J. Yang J. P. Wu X. L. Xia M. Bao M. H. (2023). Effects of Yulin Tong Bu formula on modulating gut microbiota and fecal metabolite interactions in mice with polycystic ovary syndrome. Front. Endocrinol. 14, 1122709. 10.3389/fendo.2023.1122709 PMC9939769 36814581

[B25] Wang K. Ma J. Li Y. Han Q. Yin Z. Zhou M. (2022). Effects of essential oil extracted from Artemisia argyi leaf on lipid metabolism and gut microbiota in high-fat diet-fed mice. Front. Nutr. 9, 1024722. 10.3389/fnut.2022.1024722 36407543 PMC9670120

[B26] Wang Y. H. Liang S. Xu D. S. Lin X. He C. Y. Feng Y. (2011). Effect and mechanism of senkyunolide I as an anti-migraine compound from Ligusticum chuanxiong. J. Pharm. Pharmacol. 63, 261–266. 10.1111/j.2042-7158.2010.01191.x 21235591

[B27] Wang Y. H. Hong Y. L. Feng Y. Xu D. S. Liang S. Lin X. (2012). Comparative pharmacokinetics of senkyunolide I in a rat model of migraine versus normal controls. Eur. J. Drug Metab. Ph. 37, 91–97. 10.1007/s13318-011-0073-6 22322984

[B28] Wang Z. Tang Z. Zhu W. Ge L. Ge J. (2017). Efficacy and safety of traditional Chinese medicine on thromboembolic events in patients with atrial fibrillation: a systematic review and meta-analysis. Complement. Ther. Med. 32, 1–10. 10.1016/j.ctim.2017.03.006 28619293

[B29] Wong R. S. Cheng G. Chan T. Y. (2003). Use of herbal medicines by patients receiving warfarin. Drug Saf. 26, 585–588. 10.2165/00002018-200326080-00004 12825970

[B30] Xie J. Zhao Z. Z. Li P. Zhu C. L. Guo Y. Wang J. (2021). Senkyunolide I protects against sepsis-associated encephalopathy by attenuating sleep deprivation in a murine model of cecal ligation and puncture. Oxid. Med. Cell. Longev. 2021, 6647258. 10.1155/2021/6647258 33628372 PMC7899760

[B31] Yang B. Li H. Ruan Q. Xuan S. Chen X. Cui H. (2019). Effects of gut microbiota and ingredient-ingredient interaction on the pharmacokinetics properties of rotundic acid and pedunculoside. Planta Med. 85, 729–737. 10.1055/a-0902-5300 31167298

[B32] Zhang X. Liu X. T. Kang D. Y. (2016). Traditional Chinese patent medicine for acute ischemic stroke: an overview of systematic reviews based on the GRADE approach. Med. Baltim. 95, e2986. 10.1097/MD.0000000000002986 PMC4998369 27015174

[B33] Zhang Y. Geng J. Hong Y. Jiao L. Li S. Sun R. (2019). Orally administered crocin protects against cerebral ischemia/reperfusion injury through the metabolic transformation of crocetin by gut microbiota. Front. Pharmacol. 10, 440. 10.3389/fphar.2019.00440 31114499 PMC6502977

[B34] Zuo A. Wang L. Xiao H. Li L. Liu Y. Yi J. (2011). Identification of the absorbed components and metabolites in rat plasma after oral administration of Rhizoma Chuanxiong decoction by HPLC-ESI-MS/MS. J. Pharm. Biomed. Anal. 56, 1046–1056. 10.1016/j.jpba.2011.08.010 21880453

